# Monitoring CaCO_3_ Content in Recycled Polypropylene with Raman Spectrometry

**DOI:** 10.1021/acsomega.4c00414

**Published:** 2024-05-22

**Authors:** Pixiang Wang, Dayne M. Long, Ke Zhan, Yucheng Peng, Yifen Wang, Shaoyang Liu

**Affiliations:** † Center for Materials and Manufacturing Sciences, Departments of Chemistry and Physics, Troy University, Troy, Alabama 36082, United States; ‡ College of Forestry, Wildlife and Environment, Auburn University, Auburn, Alabama 36849, United States; § Department of Biosystems Engineering, Auburn University, Auburn, Alabama 36849, United States

## Abstract

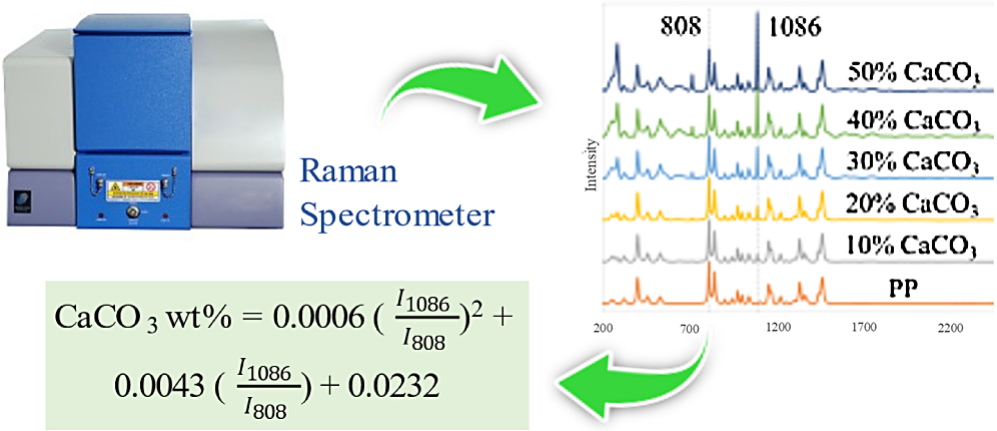

As a commonly used filler, CaCO_3_ frequently finds its way into recycled polypropylene (rPP) as a contaminant during the mechanical recycling process. Given the substantial impact of CaCO_3_ on the properties of PP materials, close monitoring of their content is important to ensure the quality of rPP. In the present work, Raman spectrometry was employed to develop a rapid, accurate, and convenient method for determining CaCO_3_ content in rPP. Partial least-squares (PLS) regression was used to construct prediction models. Various spectrum pretreatment methods, including multivariate scatter correction (MSC), standard normal variate transformation (SNV), smoothing, and first derivative, were investigated to improve the model performance. In independent validation, the optimal PLS model reached an *R*^2^ of 0.9735 and a root-mean-square error of prediction (RMSEP) of 2.7786 CaCO_3_ wt %. Furthermore, linear and second-order polynomial regressions, utilizing the intensity ratios of characteristic CaCO_3_ and PP Raman peaks, were conducted. The most effective quadratic regression curve demonstrated superior independent validation performance with an *R*^2^ of 0.9926 and an RMSEP of 1.6999 CaCO_3_ wt %. Validation with recycled PP samples confirmed that the quadratic regression was more accurate and reliable to quantify CaCO_3_ in rPP. The observed quadratic relationship between the CaCO_3_ and PP Raman peak intensity ratio and the CaCO_3_ wt % can be attributed to the significant difference in the densities of the two components. The outcomes of this research will help to facilitate the proper recycling of PP materials.

## 1 Introduction

Polypropylene (PP) is widely used in daily necessities due to its commendable mechanical properties, ease of processing, and cost-effectiveness. However, its high shrinkage rate and relatively poor impact resistance at room or low temperatures have somewhat limited its broader application. Incorporation of rigid inorganic particles is a popular approach to enhance both stiffness and toughness of plastics, which is frequently used to improve or modify the PP material properties. Among these inorganic particle fillers, calcium carbonate (CaCO_3_) is most commonly used because of its benefits on tensile and flexural modulus, availability in ready-to-use form, and economic feasibility.^1,2^ In the plastics industry alone, the global CaCO_3_ filler masterbatch market was valued at $3.5 billion in 2022 and is projected to reach $5.0 billion in 2028. Moreover, since CaCO_3_ is a naturally occurring mineral that involves almost no energy-consuming chemical reactions to produce, its carbon footprint is significantly lower than that of any synthetic polymers.^3^ As a result, PP materials incorporated with CaCO_3_ may have an evidently reduced environmental impact than neat PP and are preferred in various applications.

Recycling is a highly effective strategy to reduce the environmental impact of plastic wastes.^4,5^ This approach not only conserves precious resources but also decreases the amount of waste ending up in landfills or the natural environment. Although various recycling methods have been investigated, mechanical recycling currently retains its dominance as the more environmentally friendly and cost-efficient means of plastic recycling.^6−8^ In the mechanical recycling process, plastic wastes are collected, cleaned, melted, and regranulated for subsequent use. This process often involves blending recycled raw materials from diverse sources. Since the widespread use of CaCO_3_ as a filler in PP, it is possible to introduce it into recycled PP materials. Although the incorporation of CaCO_3_ enhances the tensile and flexural moduli of the PP composite, it significantly decreases the tensile strength and unnotched impact strength of the material.^2^ Consequently, close oversight of the CaCO_3_ content in recycled PP raw materials and products is imperative to facilitating proper processing of recycled PP and upholding product quality. Therefore, it is worthwhile to establish a convenient and accurate method to determine the CaCO_3_ content in recycled PP materials.

Spectrometric methods provide a range of rapid, nondestructive, and cost-effective tools for various polymer analyses.^9,10^ In our previous work, Raman and near-infrared (NIR) spectrometries coupled with partial least-squares (PLS) regression were employed to monitor polyethylene (PE) contamination in recycled PP.^11^ A highly accurate prediction model was established by Raman analysis. Although it is not active in the near-infrared region, CaCO_3_ shows strong peaks on the Raman spectrum, which can be used for its analysis in mixtures. Dandeu et al. applied PLS regression to the Raman spectrum and demonstrated that it permitted a good evaluation of the composition of ternary polymorph mixtures of CaCO_3_.^12^ Additionally, Park et al. utilized different CaCO_3_ Raman responses to discriminate three groups of cultured pearls.^13^ There are three naturally occurring polymorphs of CaCO_3_, i.e., calcite, aragonite, and vaterite. Among them, calcite prevails as the most abundant polymorph and serves as the predominant CaCO_3_ filler in plastics. Calcite has characteristic Raman peaks at around 1090, 715, and 285 cm^–1^.^14−16^ These peaks are different from the characteristic Raman peaks of PP. Therefore, Raman spectrometry is capable of distinguishing the two components in their mixtures, which makes it a promising tool to determine the CaCO_3_ content in recycled PP.

While linear and second-order polynomial regressions regarding the intensities of specific peaks are simple and convenient methods widely used in quantitative Raman analysis, the spectra of mixtures often present multiple overlapping bands. Thus, in addition to the height and position of the peaks, the shape and width of the bands also contain valuable information.^17^ In these cases, more sophisticated mathematical and statistical data analysis tools may allow better extraction of pertinent information from complex data. PLS regression is a popular multivariate analysis method that utilizes the entire spectral range rather than several specific positions. It is one of the favorable methods to process complicated spectral data and build reliable models to predict properties of interest.^11,18^

In this study, Raman spectrometry was employed to develop a reliable model to quantify CaCO_3_ in recycled PP. A set of blends made with commercially available PP and CaCO_3_ were used as calibration samples to establish prediction models. PLS regression with various spectrum pretreatments was investigated first. Then, linear and second-order polynomial regression analyses were assessed to identify the most effective quantification method. Recycled plastic samples were then characterized using a Raman spectrometer, and the spectra were used to validate and compare results from different models. The outcomes of this research will facilitate the proper recycling of PP materials and offer potential online quantification of CaCO_3_ in the recycling operation line.

## 2 Materials and Methods

### 2.1 Materials

A commercial grade PP with a density of 0.9 g/cm^3^ and a melt flow rate of 20 g/10 min at 230 °C/2.16 kg (Sigma-Aldrich, St. Louis, MO, USA) and a commercial grade of precipitated CaCO_3_ particles with a moisture content less than 2 wt % (Research Product International, Radnor, PA, USA) were used to prepare standard PP-CaCO_3_ blends for model calibration and independent validation.

Two types of recycled medical gown samples (rPP nos. #1 and #2) were provided by the University of Alabama at Birmingham Hospital. Our previous study identified that they were made of nonwoven PP fibers. The rPP #2 sample contains 18 wt % CaCO_3_, while the rPP #1 sample contains no CaCO_3_.^19^ The two recycled samples were used for model validation.

### 2.2 PP-CaCO_3_ Blends Preparation

The PP-CaCO_3_ blends were prepared using a C.W. Brabender mixer (CWB-2128, Hackensack, NJ, USA) equipped with two counterrotating roller blades. The thermal mixing temperature and speed were 205 °C and 60 rpm, respectively. The loading levels of CaCO_3_ were 10, 20, 30, 40, and 50 wt % based on the weight of composites (weight of CaCO_3_ plus weight of PP). In detail, PP pellets were first melted in the mixer at 205 °C for 8 min, and then CaCO_3_ particles were added to the mixer, and the mixing continued for another 5 min. After cooling for 20 min approximately, the resulting blends were scraped off and ground into pellets using a low-speed granulator (SG-2042NH, Shini Plastic Technologies Inc., Willoughby, OH, USA) with a sieve size of 3 mm. The mixture pellets were then dried and injection molded into testing specimens (63.5 mm × 12.7 mm × 3.2 mm) according to ASTM standard D256. The injection molding temperature was 195 °C, while the pressure was around 90 MPa. Neat PP specimens were also prepared with the same process.

### 2.3 Raman Spectrum Acquisition

A Raman spectrometer equipped with a 785 nm laser (MacroRAM, Horiba Scientific, Piscataway, NJ, USA) was employed to acquire the spectrum in the Raman shift range of 225–3400 cm^–1^. Each recorded spectrum represents the average of 8 individual scans. Seven replicates, collected from each calibration sample, were randomly divided into a calibration set (5 spectra) and a validation set (2 spectra). The validation set was used for independent validation. Ten replicates were collected from each recycled PP sample for additional model validation and comparison.

### 2.4 Raman Spectrum Pretreatment and Modeling

LabSpec 6 (HORIBA Scientific, Piscataway, NJ, USA) was used for spectrum smoothing with the DeNoise function (width: 3) and baseline correction with polynomial curve fitting. R program (v 4.2.2) with the mdatools package (v 0.13.1)^20^ was used for spectrum pretreatments, including Savitzkye-Golay (SG) smoothing (window size: 3, polynomial order: 1), first derivative (FD), multivariate scattering correction (MSC), and standard normal variate transformation (SNV), and PLS modeling. Excel (Microsoft, Redmond, WA, USA) was employed for linear and second-order polynomial regression analyses.

## 3 Results and Discussion

### 3.1 Raman Spectrum of CaCO_3_–PP Blends

**Figure 1:**
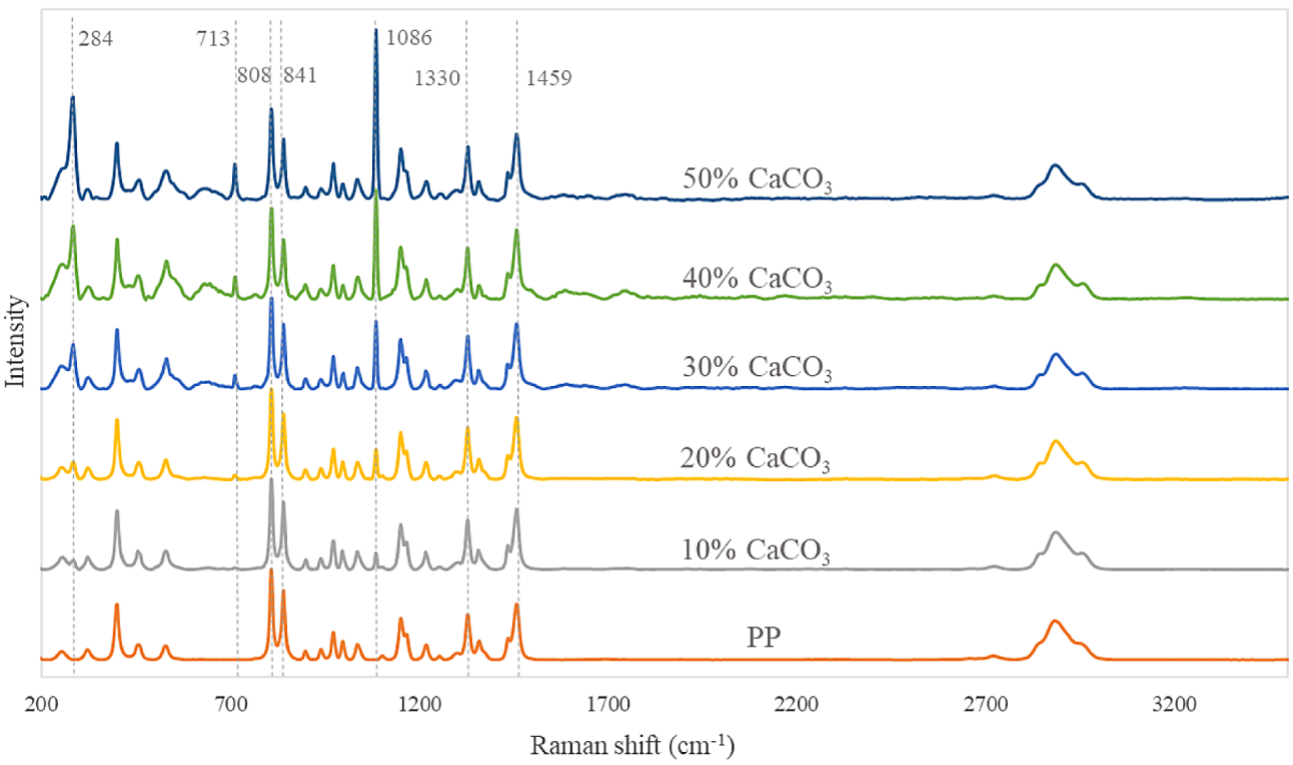
Raman spectra of different PP-CaCO_3_ blends.

The Raman spectra of the 0%–50% CaCO_3_–PP blends are shown in Figure 1. Since the collected Raman spectra had notable baseline drift and varied signal intensities, basic spectrum corrections, including denoising, baseline correction, and normalization, were performed. Good stability was observed across the replicates of the Raman spectra, indicating the relatively homogeneous nature of the samples. The CaCO_3_ shows characteristic Raman peaks at around 1086, 713, and 284 cm^–1^. The first two peaks can be assigned to symmetric stretching (*ν*_1_) and in-plane bending (ν_4_) of isolated carbonate anions, respectively.^15^ The peak at 284 cm^–1^ is related to the external vibration of the CO_3_ group that involves translatory oscillations of the group.^16^ The intensities of the above three peaks decreased with the decrease of the CaCO_3_ content and totally disappeared when the pure PP sample was analyzed. PP has four characteristic peaks with relatively high intensities in the nearby region. The peaks at 841 and 808 cm^–1^ represent the vibrations of helical molecules localized in the noncrystalline regions and the vibrations of molecules in the crystalline phase, respectively.^17^ The peaks at 1330 and 1459 cm^–1^ are associated with the twisting and in-plane bending of the CH_2_ groups in the PP molecules, respectively. The two components have clearly distinguishable peaks with relatively high intensities on Raman spectra, which suggests that Raman spectrometry is a suitable tool to determine the CaCO_3_ content in the blends.

### 3.2 Partial Least-Squares Modeling of CaCO_3_–PP Blends

The PLS modeling results with different spectrum pretreatment methods are listed in Table 1. *R*^2^, RMSEC (root-mean-square error of calibration), RMSEP (root-mean-square error of prediction), and RPD (ratio of the standard error of performance to standard deviation) were employed to evaluate the models. The RPD is calculated by dividing the standard deviation (SD) by the standard error of prediction (SEP) (RPD = SD/SEP). Better models tend to have smaller RMSEC and RMSEP, as well as larger *R*^2^ and RPD. The calibration results (*R*^2^ and RMSEC) showed that the model without spectrum pretreatment (None) had a relatively good performance with an *R*^2^ of 0.9884 and an RMSEC of 1.8419 CaCO_3_ wt %. The SNV pretreatment did not improve the model performance, while the MSC method increased the *R*^2^ to 0.9948 and lowered the RMSEC to 1.2320 wt %, suggesting the MSC pretreatment may be effective. Evident enhancement was observed when the SG-FD method was employed. The *R*^2^ increased to 0.9998 and the RMSEC decreased notably to 0.2603 wt %, indicating the SG-FD pretreatment was important to build an accurate model in this case. Slightly worse performance was obtained when the MSC or SNV method was coupled with the SG-FD pretreatment, but one less latent variable was used in the models, which might benefit the model stability in the prediction. When comparing the validation results, only the MSC-SG-FD model had a better performance than that of the model without spectrum pretreatment, demonstrating that the combination of the MSC and SG-FD pretreatments might provide the most accurate and reliable PLS model to predict CaCO_3_ content in recycled PP.

**Table 1:** PLS Modeling Results on Raman Spectrum with Different Spectrum Pretreatment Methods

		calibration		independent validation		
spectrum preatment method	LVs^a^	*R*^2^	RMSEC (wt %)	*R*^2^	RMSEP (wt %)	RPD
none	4	0.9884	1.8419	0.9486	3.8711	4.77
SNV	4	0.9882	1.8529	0.9410	4.1483	4.30
MSC	5	0.9948	1.2320	0.9325	4.4372	4.02
SG-FD	5	0.9998	0.2603	0.8984	5.4449	3.48
SNV-SG-FD	4	0.9995	0.4001	0.9261	4.6425	3.99
MSC-SG-FD	4	0.9994	0.4032	0.9735	2.7786	6.84

a LVs: number of latent variables (variables that are inferred indirectly through a mathematical model from other observable variables).

### 3.3 Linear and Second-Order Polynomial Regression Analysis of CaCO_3_–PP Blends

**Figure 2:**
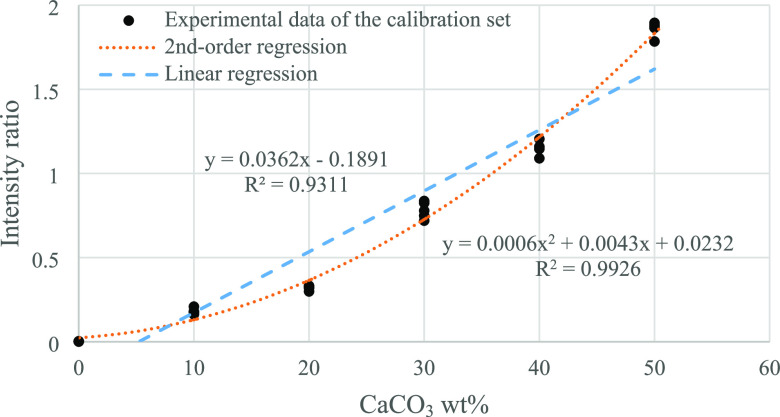
Intensity ratios of a characteristic CaCO_3_ peak (1086 cm^–1^) to a PP peak (808 cm^–1^) of different PP-CaCO_3_ blends. The fitting results are listed in the first row of Table 2.

Beside the PLS analysis, linear and second-order polynomial regressions regarding the intensity ratios of CaCO_3_/PP peaks were investigated to predict the CaCO_3_ content. Figure 2 illustrates the intensity ratios of 1068 cm^–1^ to 808 cm^–1^ peaks as a function of CaCO_3_ wt %. Since the peaks at 1068 and 808 cm^–1^ are the characteristic peaks of CaCO_3_ and PP, respectively, their ratio represents the relative content of CaCO_3_ and PP. The linear and second-order regression equations are listed below:
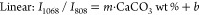

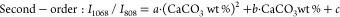
where *I* represents the intensity of the Raman spectrum at the specified Raman shift, while *m*, *a*, *b*, and *c* denote the regression coefficients.

An evident quadratic response was observed. As shown in Figure 2, the linear regression line (blue dashed line) is clearly apart from the measurement results of the calibration samples (black dots), while the second-order regression line (orange dotted line) fits the experimental data very well with a R^2^ of 0.9926.

Since CaCO_3_ has the strongest characteristic peaks at 1086 and 284 cm^–1^, and PP has the highest peaks at 808 and 1459 cm^–1^ in the nearby region, the intensity ratios of these peaks and their combinations were all evaluated in this study to find the best quantification method. The calibration and independent validation results are listed in Table 2. The second-order regression had notably higher *R*^2^ and lower RMSEP in all cases, confirming the quadratic relationship between the intensity ratio and the CaCO_3_ wt %. Among the methods, the ratios of (1086 + 284)/808 and (1086 + 284)/(808 + 1459) showed the highest *R*^2^s, but their RMSEPs were the largest, suggesting possible overfitting. The ratios of 1086/808, 1086/(808 + 1459), and (1086 + 284)/1459 had *R*^2^s above 0.99 and RMSEPs below 1.75 wt %, which demonstrated that they might be the most accurate methods to predict CaCO_3_ content with the second-order regression. Surprisingly, their RMSEPs were much lower than those from the PLS modeling in combination with the MSC-SG-FD method, implying that the second-order regression method may be more suitable than the PLS modeling in this study.

**Table 2:** Linear and Second-Order Polynomial Regression Results Based on the Intensity Ratios of CaCO_3_/PP Peaks

	linear		second-order polynomial	
relative intensity ratio of CaCO_3_ peak(s) to PP peak(s)	calibration, *R*^2^	independent validation RMSEP (wt %)	calibration, *R*^2^	independent validation RMSEP (wt %)
1086/808	0.9311	3.1327	0.9926	1.6999
1086/(808 + 1459)	0.9305	3.2819	0.9916	1.7114
1086/1459	0.9285	4.3946	0.9889	1.7914
(1086 + 284)/808	0.9356	10.1534	0.9932	7.4135
(1086 + 284)/(808 + 1459)	0.9360	10.2916	0.9930	7.5401
(1086 + 284)/1459	0.9350	4.0889	0.9914	1.7350
284/808	0.9388	3.7590	0.9896	2.2038
284/(808 + 1459)	0.9400	3.7490	0.9904	2.0809
284/1459	0.9404	3.7798	0.9904	1.9528

### 3.4 Model Validation with Recycled PP Materials

Since the purpose of this study was to monitor CaCO_3_ content in recycled plastics, two recycled PP samples with known CaCO_3_ content from our previous work were employed to validate the two best PLS models (None and MSC-SG-FD) and the three best second-order regression curves.^19^ The results are listed in Table 3. The prediction results of the two PLS models were very poor. It was noticed that some pigments/additives in the recycled samples caused additional peaks on the Raman spectrum, which could affect the model prediction. Therefore, several strategies were tested to optimize the spectrum range selection for PLS modeling. However, no meaningful improvement was made. The results suggest that PLS modeling may not be suitable for this task, although it is a popular and capable method for various quantitative spectrometric analyses.

**Table 3:** Model Validation Results with Recycled PP Samples

		rPP #1 (0 CaCO_3_ wt %)		rPP #2 (18 CaCO_3_ wt %)	
model		RMSEP	prediction result^a^	RMSEP	prediction result^a^
PLS	None	11.4783	9.9 ± 5.9	5.4670	13.2 ± 2.6
	MSC-SD-FD	28.8772	-27.3 ± 9.4	7.8720	11.3 ± 4.1
second-order	1086/808	3.2396^b^	-3.1 ± 1.0^b^	2.5515	17.6 ± 2.7
	1086/(808 + 1459)	3.5083^b^	-3.5 ± 0.0^b^	2.6659	17.8 ± 2.8
	(1086 + 284)/1459	4.0319^b^	-3.8 ± 1.5^b^	2.8889	17.1 ± 2.9
second-order (constrained through the origin)	1086/808	1.2270	0.1 ± 1.3	2.4639	17.7 ± 2.6
	1086/(808 + 1459)	0.9619	0.1 ± 1.0	2.5731	17.9 ± 2.7
	(1086 + 284)/1459	2.5669	2.1 ± 1.5	4.7209	21.8 ± 2.9

a Expressed as average ± standard deviation (*n* = 10).

b Some of the experimental responses were lower than the lowest point of the fitting quadratic curve, and the prediction results were forced to be the lowest possible prediction value.

On the other hand, the prediction results based on the second-order regression were much better. The predicted CaCO_3_ contents were close to the true values. The curve from the ratio of the highest peaks of the two components (1086/808) had a slightly better performance than that of the other two. It was noticed that all the prediction results for rPP #1, which had no CaCO_3_, were negative. Given that the CaCO_3_ content cannot fall below zero, and theoretically, the Raman intensity ratio should be zero when the CaCO_3_ content is zero, the second-order fitting curve was constrained to pass through the origin, and the regressions were carried out again. This adjustment should help reduce prediction errors near the zero point, which may improve the prediction results for rPP #1. The RMSEPs of independent validation of the 1086/808 and 1086/(808 + 1459) fitting curves through the origin only slightly increased to 1.7608 and 1.7888, respectively, implying they maintained overall good performance. But that of the new (1086 + 284)/1459 curve jumped to 4.9757, suggesting the prediction error of the last curve might rise dramatically. Upon validation with the rPP samples, a significant enhancement in prediction accuracy was observed for rPP #1 across all curves. However, the (1086 + 284)/1459 curve displayed a noteworthy decline in overall performance, consistent with the independent validation results. The 1086/808 and 1086/(808 + 1459) fitting curves through the origin achieved comparable high accuracy for both rPP samples. The utilization of second-order regression appears to be a more appropriate approach for predicting CaCO_3_ content in recycled PP using Raman spectra. Notably, this regression method, relying on the intensities of only two or three peaks, may have an enhanced ability to resist interfering peaks from pigments and other additives in recycled materials. Considering its ease of operation, the 1086/808 curve may be the most effective method for monitoring the CaCO_3_ content in recycled PP.

The reason for the observation of an apparent quadratic response was further investigated. In Raman spectrometry analysis, the spectrometer applies a strong laser beam to the sample to acquire analytical signals. When the photons in the laser beam interact with the analyte, inelastic scattering can happen. The Raman spectrometer collects the inelastically scattered photons to obtain the Raman spectrum. Therefore, direct interaction between the photon and the analyte is required to generate a Raman signal.

When a laser beam is applied to the PP-CaCO_3_ composite, there are three situations:

a) If a CaCO_3_ particle is on the surface of the sample, only the CaCO_3_ signal will be observed.

b) If there’s no CaCO_3_ particle in the light path or the particle is embedded deeply in the composite, only the PP signal will be obtained.

c) If a CaCO_3_ particle is embedded near the surface, both PP and CaCO_3_ signals will be generated. The relative intensities of the two signals are a function of the depth of the CaCO_3_ particle.

The (c) situation is complicated. Fortunately, the laser beam of a Raman spectrometer can penetrate only a shallow layer of the sample, typically several micrometers. The chance of CaCO_3_ particles embedded in such a thin layer is low. Therefore, the overall Raman signal is predominantly influenced by situations (a) and (b). As a result, the relative intensity of the CaCO_3_ to PP signals primarily hinges on how much space the two components occupy, or, in other words, the volume ratio of the two components, rather than the weight percentage of CaCO_3_.

Due to the significant difference in density between PP (ca. 0.9 g/cm^3^) and CaCO_3_ (ca. 2.7 g/cm^3^), their weight and volume ratios are not proportional. Assuming the mass of the composite is *m*, the relationship between the weight and volume percentages of CaCO_3_ in PP- CaCO_3_ composite can be calculated below:
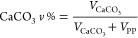

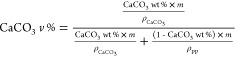
where *V* is the volume of the corresponding component and ρ represents density. After rearrangement, the following equation is obtained:
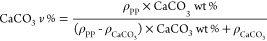


So,
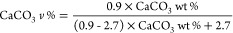

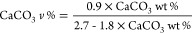


**Figure 3:**
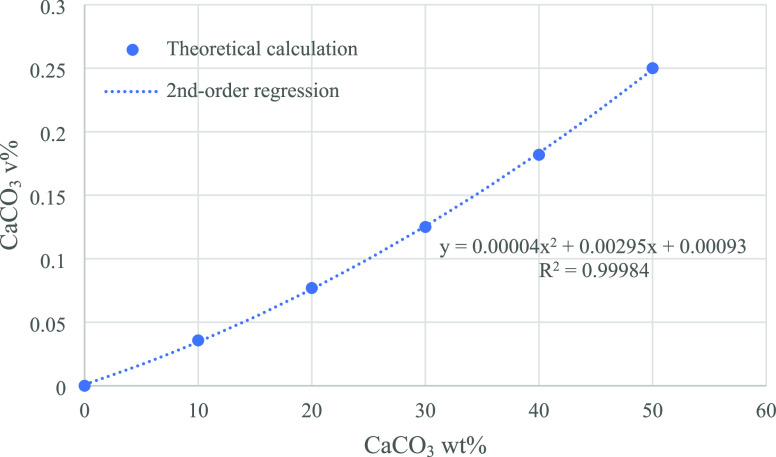
Relationship between the weight and volume percentages of CaCO_3_ in PP-CaCO_3_ blends.

Figure 3 illustrates the correlation between the weight and volume percentages of CaCO_3_ in the 0–50 wt % range, as calculated by the aforementioned equation (represented by blue dots). A quadratic curve (depicted as a dotted line) aligns remarkably well with the theoretical points (*R*^2^ = 0.9998). Given that the relative intensity of the CaCO_3_ to PP signals is proportional to the CaCO_3_ volume fraction, an apparent quadratic relationship between the Raman signal ratio and the CaCO_3_ wt % was observed.

## 4 Conclusions

Raman spectrometry is an effective tool to quantify the CaCO_3_ content in recycled PP. PLS regression coupled with various spectrum pretreatment methods, e.g., MSC, SNV, and first-derivative, were employed to construct prediction models. The optimal model reached an independent validation RMSEP of 2.7786 CaCO_3_ wt %. In addition, linear and second-order polynomial regressions relying on the CaCO_3_ and PP Raman peak intensity ratios were carried out. The best second-order regression model demonstrated a superior independent validation RMSEP of 1.6999 CaCO_3_ wt %. The subsequent validation using recycled PP samples confirmed that the quadratic regression was more accurate and reliable to determine CaCO_3_ content in recycled PP. The apparent quadratic relationship between the CaCO_3_ and PP Raman peak intensity ratio and the CaCO_3_ wt % can be attributed to the significant difference in the densities of the two components.
